# Giant beaver palaeoecology inferred from stable isotopes

**DOI:** 10.1038/s41598-019-43710-9

**Published:** 2019-05-09

**Authors:** Tessa Plint, Fred J. Longstaffe, Grant Zazula

**Affiliations:** 1 Department of Earth Sciences, The University of Western Ontario, London, Ontario Canada; 2 Present Address: The Lyell Centre, School of Energy, Geoscience, Infrastructure and Society, Heriot-Watt University, Edinburgh, United Kingdom; 3 Yukon Palaeontology Program, Government of Yukon, Whitehorse, Yukon Territory Canada; 4 Research and Collections, Canadian Museum of Nature, PO Box 3443, Station D, Ottawa, ON K1P 6P4 Canada

**Keywords:** Biogeochemistry, Palaeoecology

## Abstract

This is a multi-individual (n = 11), stable carbon and nitrogen isotope study of bone collagen (*δ*^13^C_col_ and *δ*^15^N_col_) from the giant beaver (genus *Castoroides*). The now-extinct giant beaver was once one of the most widespread Pleistocene megafauna in North America. We confirm that *Castoroides* consumed a diet of predominantly submerged aquatic macrophytes. These dietary preferences rendered the giant beaver highly dependent on wetland habitat for survival. *Castoroides*’ *δ*^13^C_col_ and *δ*^15^N_col_ do not support the hypothesis that the giant beaver consumed trees or woody plants, which suggests that it did not share the same behaviours as *Castor* (*i*.*e*., tree-cutting and harvesting). The onset of warmer, more arid conditions likely contributed to the extinction of *Castoroides*. Six new radiocarbon dates help establish the chronology of the northward dispersal of the giant beaver in Beringia, indicating a correlation with ice sheet retreat.

## Introduction

### The Pleistocene giant beaver

The one hundred kilogram giant beaver (genus *Castoroides*) inhabited North America throughout the mid- to late Pleistocene^1–3^. The giant beaver went extinct along with dozens of other genera during the late Pleistocene megafauna extinction^4,5^. The underlying mechanism(s) behind this global extinction remains contested, though it is often attributed to a combination of climate change and anthropogenic impacts^6–12^. Current information regarding giant beaver ecology is insufficient to evaluate various hypotheses posited to be responsible for *Castoroides*’ extinction. The last appearance date of 10,150 ± 50 years BP for a *Castoroides ohioensis* specimen from Wayne County, New York demonstrates that it was also one of the late surviving members of the Pleistocene megafauna community in North America^6^. Here we use stable isotopes to explore the ecology of *Castoroides*, which allows us to better understand its diet, its impact on the surrounding Pleistocene landscape, and the mechanisms that led to its demise. Previous models of *Castoroides*’ diet and habitat preference are based on skeletal morphology, fossil depositional environment, and the behaviour of the only remaining extant member of the Castoridae family, *Castor*.

### Extant analogue species

The short limbs and bulky body of *Castoroides* made it poorly adapted to a life spent predominantly on land and it must have relied on access to the water for shelter from terrestrial predators^13^. Previous studies based on stable isotope analysis of single specimens suggest that the giant beaver consumed aquatic vegetation, and thrived in mid-latitude regions of North America during warm, strongly seasonal conditions between 125,000 and 75,000 BP^14,15^. Other studies hypothesize that the giant beaver was a relatively cold-tolerant species, preferentially consumed emergent macrophytes, and lived in ponds and shallow lakes bordered by marshlands^16–19^. The presence of giant beaver fossils in high latitude regions such as Alaska and Yukon Territory confirm it could persist through harsh arctic climatic and low light conditions in the winter. It is unknown if *Castoroides* shared any behavioural characteristics with the genus *Castor*, and the possibility that the giant beaver engineered its habitat by constructing dams or lodges remains a topic of debate^1,17,20–24^.

Two competing models of *Castoroides* diet and behaviour have emerged based on extant North American semi-aquatic rodents with well-documented ecologies and distinctly different impacts on the surrounding ecosystem^14,19,25–27^. The first model predicts that *Castoroides* filled an econiche similar to that of the muskrat (*Ondatra zibethicus*), as a semi-aquatic rodent that primarily consumed submerged and emergent freshwater macrophytes in calm wetlands with an expansive littoral zone^14,19–21^. Such feeding would have kept shallow waterways clear of excess macrophyte growth and promoted aquatic biotic diversity. The second model suggests that *Castoroides* filled an econiche similar to that of the extant North American beaver (*Castor canadensis*), as a semi-aquatic rodent that consumed both terrestrial and aquatic vegetation, and practiced tree-cutting behaviour for the purpose of lodge and dam building^25^. The bulk of its diet would have been foliage from deciduous trees and its engineering habits would have promoted local biotic diversity and significantly impacted the hydrological patterns of the Pleistocene landscape.

Problematic to the latter hypothesis is that there are neither confirmed discoveries of dam or lodge structures built by giant beavers, nor substantiated evidence of Pleistocene-era cut wood that matches the occlusal surface size and angle of *Castoroides* incisors^28,29^. The angle of protrusion of the incisors from the skull and their lack of a thin, chisel-like edge rendered giant beaver incisors ineffective tools for cutting down trees^21^. This has not stopped speculation that masses of branches discovered in proximity to *Castoroides* fossils in Pleistocene sediments were built by the giant beaver^25^. In addition, tree harvesting behaviours have been present in certain branches of the Castoridae family since the Miocene^30,31^.

### Stable carbon and nitrogen isotopes and dietary mixing models

The stable carbon and nitrogen isotope compositions (*δ*^13^C and *δ*^15^N) of an animal’s body tissues closely reflect those of its diet, with adequately known isotopic discrimination factors occurring between each trophic level^32–34^. Thus, the isotopic composition of modern and ancient bone collagen serves as a useful proxy for diet^35–38^. To correctly assess trophic position and forage preference, however, potential dietary sources must be established^39^.

**Figure 1:**
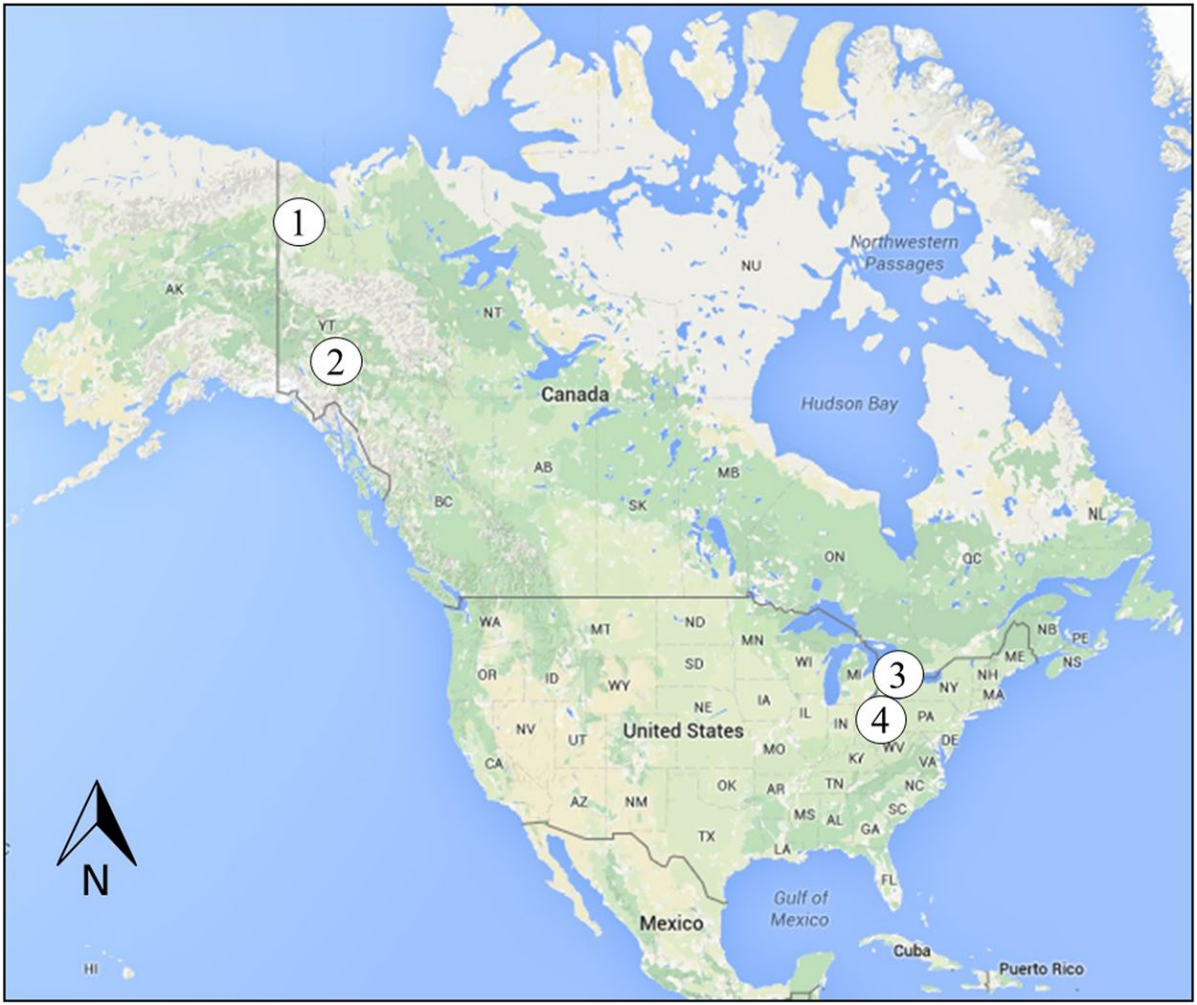
Terrain map of North America indicating sample collection sites. 1 – Old Crow, Yukon Territory, Canada. 2 – Whitehorse, Yukon Territory, Canada. 3 – Pinery Provincial Park and London Area, Ontario, Canada. 4 – Ohio, United States of America. Map data from Google, INEGI, 2019.

Here, *Castoroides*, *O*. *zibethicus* and *C*. *canadensis* bone collagen carbon and nitrogen isotope compositions (*δ*^13^C_col_ and *δ*^15^N_col_) are assessed within the context of modern terrestrial and freshwater plant carbon and nitrogen isotope signatures. Modern plant samples were collected from shallow lakes, marshes, and creeks located in the Great Lakes region of southwestern Ontario (Pinery Provincial Park and the London area) and Yukon Territory (Whitehorse and Old Crow Basin), Canada, whereas *Castoroides* specimens originate from sites in Yukon Territory, Canada, and Ohio, USA (Fig. 1). The isotopic data were incorporated into a statistically-based dietary mixing model (SIAR V4: Stable Isotope Analysis in R. An Ecologists’ Guide) to determine both forage choice and relative input proportions for each rodent species^40^. This allows us to determine the degree of dietary overlap amongst rodent species, *Castoroides*’ level of dependence on aquatic habitat space, and the viability of using *C*. *canadensis* or *O*. *zibethicus* as extant analogues in palaeoecological models incorporating the giant beaver. A series of new radiocarbon dates obtained from *Castoroides* bone collagen from Yukon Territory and Ohio are also reported to evaluate the chronology of regional extirpation.

### Stable carbon and nitrogen isotopes of freshwater plants

Carbon and nitrogen dynamics are complex in freshwater systems. As a result, freshwater plant, or macrophyte *δ*^13^C and *δ*^15^N vary widely^39,41–43^. Plant physiology and local environmental conditions account for broad-scale isotope variability; site specific characteristics (*i*.*e*., wetland habitat type, water turbulence, latitude, local geology, water pH, water temperature) also impact the isotopic composition of bioavailable carbon and nitrogen sources. Habitat division within a wetland also has a strong impact on macrophyte *δ*^13^C and *δ*^15^N^39^. Plant functional groups used here (terrestrial trees and shrubs, emergent macrophytes, floating macrophytes, and submerged macrophytes) were categorized not by taxonomic relationship, but by the plant’s exposure to the atmosphere, the water column, and/or substrate. These three media determine which sources of bioavailable carbon and nitrogen the plant can access, and ultimately control plant *δ*^13^C and *δ*^15^N.

### Complexities arising from the use of modern plants in palaeodietary studies

Anthropogenic influence on global or regional carbon and nitrogen isotope compositions at the base of the food web is one concern when utilizing modern plant *δ*^13^C and *δ*^15^N as a basis for palaeodietary interpretations. Photosynthetic pathway is the dominant control on plant *δ*^13^C and atmospheric CO_2_ is the primary carbon source for photosynthetic organisms. A Suess effect correction can be applied to modern *δ*^13^C data to account for the recent addition (primarily through the burning of fossil fuels) of ^12^C-enriched CO_2_ to the atmosphere^44^.

Assessment of the nitrogen isotope baseline is more complicated as it is specific to region and ecosystem. Anthropogenic activities (particularly land development and agricultural practices) can contribute nitrogen sources that enter the local environment and impact the nitrogen isotope composition of primary producers. This effect is particularly pronounced in modern wetland habitats^45^.

There is also evidence that regional nitrogen isotope baselines of some ecosystems changed over the course of the Quaternary. For instance, the dynamic of the nitrogen cycle and the isotopic composition of the nitrogen baseline in grasslands of Yukon Territory have changed between the Late Pleistocene and the present^46^. There, Mammoth Steppe plant macrofossil *δ*^15^N was on average ~2.8‰ higher than that of modern grassland plants collected from same location^46^. This ^15^N-enrichment at the base of the food web was incorporated into successive trophic levels and contributed to unusually high *δ*^15^N_col_ of Mammoth Steppe grassland herbivores (such as the Arctic ground squirrel and the woolly mammoth)^38,46^. The plant macrofossil ^15^N-enrichment was likely the result of an arid climate and a more open nitrogen cycle^47^. It is less likely, however, that Pleistocene wetland ecosystems experienced such a shift in *δ*^15^N.

The ^15^N-enrichment of the nitrogen baseline observed in the Mammoth Steppe grasslands was induced by factors that would not have been nearly as pronounced in Pleistocene wetland ecosystems, or wetter, forested ecosystems with lower megafauna population densities. Late Pleistocene mastodon and giant beaver populations, for example, were contemporary in Yukon Territory and in the Great Lakes region of North America^48,49^. These species both showed preference for the same type of habitat (open mix-forest interspersed with wetlands). Mastodon were browsers and their collagen is significantly depleted of ^15^N relative to Mammoth Steppe fauna that were graminoid and forb specialists (*e*.*g*., in North America, average *δ*^15^N_col_ of mastodon is ~4‰ lower than mammoth)^38,50,51^. In short, while plant enrichment in ^15^N arising from aridity and associated shifts in the nitrogen cycle can profoundly affect grassland plants, we posit that pronounced changes are much less likely in plants from forested and wetland habitats. In the absence of unaltered ancient equivalents, modern plants from uncontaminated wetlands or wetter, forested habitats can therefore provide an adequate, if not ideal, proxy for incorporation into isotopic models that simulate Pleistocene dietary preferences.

In addition, the relative nitrogen isotope pattern of each plant functional group should remain the same, despite changes in nitrogen baseline *δ*^15^N. Aquatic plants (submerged, emergent, and floating macrophytes), for example, should have, on average higher *δ*^15^N than terrestrial plants on account of their access to more ^15^N-enriched sources of bioavailable nitrogen.

## Results

### Radiocarbon dates

Six new radiocarbon dates obtained from *Castoroides* bone and dentin collagen are reported in Table 1. Bone collagen from *Castoroides* that lived at high latitudes north of the Arctic Circle yielded dates that are analytically non-finite or so close to the analytical limit of radiocarbon dating (~45,000 ^14^C years BP) that they can be treated as non-finite ages. An additional summary of *Castoroides* radiocarbon dates from the literature is presented in Table 2.

**Table 1:** Radiocarbon ages for *Castoroides* specimens (bone and tooth dentin collagen) analyzed in this study.

Project sample ID	Institute accession ID	Specimen locality	^14^C age (BP)	±	AMS Lab code
C3-TP2014	CMN 18306	Old Crow Basin, Yukon Territory	>44,600		UCIAMS151528
C4-TP2014	CMN 18707	Old Crow Basin, Yukon Territory	>43,700		UCIAMS151529
C6-TP2014	CMN 14711	Old Crow Basin, Yukon Territory	44,600	2600	UCIAMS151530
C8-TP2014	CMN 33640	Old Crow Basin, Yukon Territory	>42,200		UCIAMS151531
C11-TP2014	OHS N9087	Williams County, Ohio	11,961	80	AA105557
C22-TP2014	OHS N8739	Harmony Township, Ohio	11,168	53	AA105559

**Table 2:** Literature summary of *Castoroides* radiocarbon dates.

Taxon	Specimen locality	Tissue type	^14^C age (BP)	±	AMS Lab Code	Reference
*Castoroides ohioensis*	Fairy Hole Rockshelter, Allamuchy Township, Warren Co., New Jersey	Molar (collagen)	11,140	30	UGAMS-16240	Boulanger *et al*.^65^
*Castoroides ohioensis*	Big Brook locality, Holmdel Township, Monmouth Co., New Jersey	Molar (bioapatite)	13,210	30	UGAMS-18142	Boulanger *et al*.^65^
*Castoroides ohioensis*	Dutchess Quarry Cave 8, Orange Co., New York	Molar (collagen)	11,670	70	NSRL-1513	Steadman *et al*.^96^
*Castoroides*	Clyde, Wayne Co., New York	Bone (collagen)	10,150	50	OS-73632	Fernac and Kozlowski^97^
*Castoroides*	Kansas River, Johnson Co., Kansas	Mandible (collagen)	12,150	80	CAMS-20004	McDonald and Glotzhober^66^
*Castoroides*	Mississippi River, Ramsey Co., Minnesota	Bone (collagen)	10,320	250	not provided	Erickson^98^
*Castoroides*	Clear Creek, Fairfield Co., Ohio	Mandible (collagen)	12,040	35	UCLAMS-11219	McDonald and Glotzhober^66^
*Castoroides*	Sheriden Pit, Wyandott Co., Ohio	Bone (collagen)	10,850	60	CAMS-26783	Tankersley and Landefeld^99^

### Collagen preservation

Collagen yield (wt. %), atomic C:N ratio, and carbon and nitrogen contents (wt. %) were used to assess collagen preservation in all three rodent species (Tables 3 to 5). All *Castoroides* specimen parameters are within the accepted range for archaeological skeletal material from temperate or polar regions (collagen yield > 1%; %C = 34.8 ± 8.8%; %N = ~11–16%; C:N = 3.1–3.5)^52^.

**Table 3:** *Castoroides* specimen information and stable isotope results. Values in bold indicate mean and 1 SD where duplicate analyses were completed for the same specimen.

Project sample ID	Institute accession ID	Specimen locality	Skeletal element	*δ*^13^C_col_ (‰, VPDB)	*δ*^15^N_col_ (‰, AIR)	C (wt. %)	N (wt. %)	Collagen yield (wt. %)	Atomic C:N
C1-TP2014	CMN 16657	Old Crow Basin, YT	Humerus	−21.2	+6.3	41.7	15.6	1.4	3.1
C3-TP2014	CMN 18306	Old Crow Basin, YT	Pelvis	−**19**.**1** ± **0**.**3**	+**1**.**9** ± **0**.**1**	41.5	15.2	1.4	3.2
C4-TP2014	CMN 18707	Old Crow Basin, YT	Tibia	−**10**.**7** ± **0**.**2**	+**5**.**7** ± **0**.**1**	41.8	15.5	3.6	3.1
C5-TP2014	CMN no ID	Old Crow Basin, YT	Femur	−18.5	+7.7	41.2	15.7	Data not provided*	3.1
C6-TP2014	CMN 14711	Old Crow Basin, YT	Humerus	−16.0	+6.0	33.1	11.9	1.1	3.2
C7-TP2014	CMN 14781	Old Crow Basin, YT	Long bone diaphysis	−14.0	+7.4	39.6	14.5	Data not provided*	3.2
C8-TP2014	CMN 33640	Old Crow Basin, YT	Humerus	−**12**.**4** ± **0**.**2**	+**6**.**2** ± **0**.**0**	39.7	14.4	2.4	3.2
C9-TP2014	CMN 43178	Old Crow Basin, YT	Femur	−21.2	+6.8	42.7	15.5	Data not provided*	3.2
C10-TP2014	OHS N9109	Clear Creek Township, OH	Mandible	−**20**.**2** ± **0**.**1**	+**5**.**6** ± **0**.**1**	35.5	12.6	3.1	3.3
C11-TP2014	OHS N9087	Williams County, OH	Mandible	−**20**.**6** ± **0**.**1**	+**4**.**5** ± **0**.**1**	40.0	14.3	11.2	3.3
C22-TP2014	OHS N8739	Harmony Township, OH	Incisor (dentin)	−19.5	+5.4	37.4	13.5	3.8	3.2

*Collagen extraction performed at the Keck Carbon Cycle AMS Facility at the University of California, Irvine for radiocarbon dating. Remaining collagen material was returned for stable isotope analysis at the University of Western Ontario.

**Table 4:** *Ondatra zibethicus* specimen information and stable isotope results. Values in bold indicate mean and 1 SD when duplicate analyses were performed for the same specimen. Specimens collected between August 2013 and August 2014. Stable carbon isotope data are not corrected for the Suess effect.

Project sample ID	Specimen locality	Skeletal element	*δ*^13^C_col_ (‰, VPDB)	*δ*^15^N_col_ (‰, AIR)	C (wt. %)	N > ((wt. %)	Collagen yield (wt. %)	Atomic > C:N
M1-TP2013	London, ON	Mandible	−22.7	+5.0	41.1	15.5	8.6	3.1
M3-TP2014	Old Crow Flats, YT	Mandible	−13.6	+5.3	42.6	14.9	11.8	3.3
M4-TP2014	Old Crow Flats, YT	Mandible	−14.2	+2.2	42.1	16.1	12.2	3.0
M5-TP2014	Old Crow Flats, YT	Mandible	−17.5	+3.8	42.5	14.9	10.8	3.3
M6-TP2014	Old Crow Flats, YT	Mandible	−22.0	+5.9	42.5	16.1	13.3	3.1
M7-TP2014	Old Crow Flats, YT	Mandible	−15.1	+6.1	42.6	16.2	11.3	3.1
M8-TP2014	Old Crow Flats, YT	Mandible	−14.6	+3.9	43.2	15.1	13.4	3.3
M9-TP2014	Old Crow Flats, YT	Mandible	−14.3	+6.6	42.8	14.3	13.3	3.5
M10-TP2014	Old Crow Flats, YT	Mandible	−15.0	+5.3	43.2	16.4	12.7	3.1
M11-TP2014	Old Crow Flats, YT	Mandible	−12.6	+5.7	43.6	16.7	13.5	3.0
M13-TP2014	Old Crow Flats, YT	Mandible	−**10**.**7** ± **0**.**1**	+**6**.**0** ± **0**.**0**	42.6	14.9	13.7	3.3
M14-TP2014	Old Crow Flats, YT	Mandible	−11.7	+4.7	42.8	15.0	12.8	3.3

**Table 5:** *Castor canadensis* specimen information and stable isotope results. Specimens collected between August 2013 and August 2014. Stable carbon isotope data are not corrected for the Suess effect.

Project sample ID	Specimen locality	Skeletal element	*δ*^13^C_col_ (‰, VPDB)	*δ*^15^N_col_ (‰, AIR)	C (wt. %)	N (wt. %)	Collagen yield (wt. %)	Atomic C:N
B1-TP2013	Inverhuron, ON	Tibia	−23.6	+7.1	43.9	16.9	12.0	3.0
B2-TP2013-A	Dawson City, YT	Metapodial	−24.0	+3.4	44.8	16.9	10.2	3.1
B3-TP2014-1	New Liskeard, ON	Mandible	−23.7	+2.0	43.6	16.6	14.4	3.1
B4-TP2014-1	New Liskeard, ON	Mandible	−23.1	+9.5	43.6	16.7	14.7	3.0
B5-TP2014	New Liskeard, ON	Mandible	−23.2	+8.0	42.7	16.3	14.8	3.1
B6-TP2014-1	Whitehorse, YT	Mandible	−23.7	+2.0	43.7	16.8	16.7	3.0
B7-TP2014	Whitehorse, YT	Mandible	−23.3	+1.4	43.1	16.6	16.6	3.0
B8-TP2014-1	Whitehorse, YT	Mandible	−23.7	+2.2	42.3	16.2	16.3	3.0

### Stable isotopes of bone and dentin collagen

*Castoroides* (n = 11) *δ*^13^C_col_ ranges from −21.2 to −10.9‰, with a mean of −17.6‰; *Castoroides δ*^15^N_col_ ranges from +1.9 to +7.7‰, with a mean of +5.8‰ (Table 3). Extant *O*. *zibethicus* (Table 4) and *C*. *canadensis* (Table 5) show distinctly different isotopic patterns. The carbon and nitrogen isotope compositions of muskrat bone collagen closely resembles that of *Castoroides*; *O*. *zibethicus* (n = 12) *δ*^13^C_col_ = −22.7 to −10.7‰, with a mean of −15.3‰, and *O*. *zibethicus δ*^15^N_col_ = +2.2 to +6.6‰, with a mean of +5.1‰. *C*. *canadensis* (n = 8) *δ*^13^C_col_ varies very little, ranging from −24.0 to −23.2‰, with a mean of −23.5‰. *C*. *canadensis δ*^15^N_col_ displays more variation, ranging from +1.4 to +9.5‰, with a mean of +4.5‰.

### Stable isotopes of plants

Modern plant sample *δ*^13^C and *δ*^15^N are listed in Tables A and B in Supplementary Information File SI1. A summary of plant functional group *δ*^13^C and *δ*^15^N mean and range are presented in Table C in Supplementary Information File SI1. Generally, macrophytes have higher *δ*^13^C and *δ*^15^N than terrestrial plants. Submerged macrophytes display a bimodal distribution of *δ*^13^C (ranging from −41 to −13‰). These isotopic patterns are demonstrated in Figure A in Supplementary Information File SI1.

## Discussion

Our terrestrial and freshwater plant *δ*^13^C and *δ*^15^N results are consistent with those of other studies from comparable regions^53,54^. While we do not have the perfect stable isotope dietary mixing model (*i*.*e*., one comprising coeval plant macrofossil *and* faunal material from the same site), modern plant sampling sites were purposefully chosen that had minimal observed or documented anthropogenic impact. Anthropogenic impact on the nitrogen cycle and isotopic composition of wetland habitats in Pinery Provincial Park is minimal^55^. Plant samples from London, Ontario (n = 9) were collected from non-urban areas. While we cannot rule out that the *δ*^15^N of these nine samples may have been impacted by agriculture, chemical fertilizer (*δ*^15^N ≈ 0‰) is dominantly used in this region, which constrains possible variation from that source^56^. Yukon Territory has very low human population density (<36,000), and minimal industrial and agricultural activities^57^. As such, we expect minimal influence on bioavailable sources of nitrogen (*i*.*e*., septic effluent, chemical, manure) and *δ*^15^N of plants collected around Whitehorse and Old Crow.

In addition, the majority of the plant taxa included in this study (see plant taxonomic information in Supplementary Information File SI1 Tables A and B) are known to have existed in North America during the late Pleistocene (refer to^19,58–60^ for examples of plant macrofossil and pollen records recovered from Pleistocene sites from Beringia and the Great Lakes region). Plants sampled also included tree and macrophyte species documented as part of the diet of extant *C*. *canadensis* and *O*. *zibethicus*^61–64^. All this said, a great opportunity remains to further test the ideas presented here through isotopic analysis of contemporary Pleistocene plant macrofossils associated with *Castoroides*.

Overall, collagen yields from giant beaver specimens were relatively low. This was particularly surprising for *Castoroides* specimens collected from Old Crow, Yukon Territory. Pleistocene faunal remains from Beringian deposits typically have excellent organic molecule preservation. We propose that the low collagen yields are in fact linked to giant beaver habitat preference. *Castoroides* remains are most commonly recovered from active or ancient wetland environments^19,65,66^. In addition, specimens from the Canadian Museum of Nature (CMN) collections included in this study were recovered from the banks of the Porcupine River, Yukon Territory, as reported by C.R. Harington. Given its semi-aquatic nature, most giant beavers likely died in close proximity to a wetland habitat. The composite nature of bone (bioapatite crystals interlocked with strands of collagen protein) results in a structure where the mineral component protects the protein component (particularly from detrimental microorganism enzyme activity)^67^. Exposure to water in a *post-mortem* depositional environment, however, accelerates bone diagenetic processes. The deposition of giant beaver skeletal material in an aqueous environment accelerates dissolution of the bioapatite, after which it can no longer protect the organic fraction and collagen loss is more likely to occur.

A Suess effect correction of 2.1‰ was applied to modern rodent *δ*^13^C_col_ and to the *δ*^13^C of plants collected in 2014. A correction of 1.7‰ was applied to the *δ*^13^C of plants collected in 2000. Refer to Supplementary Information File SI1 Table A and B for plant sample collection dates. The corrections were calculated using data available through the Scripps CO_2_ monitoring program and the *δ*^13^C of late Pleistocene atmospheric CO_2_ described in Schmitt *et al*.^68,69^. The Suess effect correction was applied when modern *δ*^13^C data were incorporated into the SIAR mixing model.

**Figure 2:**
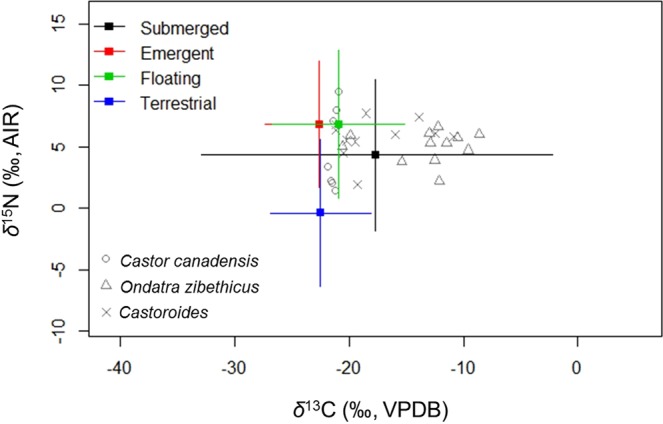
Stable carbon and nitrogen isotope compositions of rodent collagen and vegetation. Rodent *δ*^13^C_col_ and *δ*^15^N_col_ are corrected for trophic enrichment factors to render them comparable to the four plant functional groups (represented by their mean and a range of 2 SD) that may have comprised their diet. Carbon isotope data for modern plants and rodents are corrected for the Suess effect to enable direct comparison with results for *Castoroides*.

Based on existing literature, collagen-diet offsets of +4.2‰ and +3.0‰ were utilized for *δ*^13^C and *δ*^15^N, respectively^33,70–72^. Collectively, the corrections for the Suess effect and dietary isotopic discrimination render the rodent *δ*^13^C_col_ and *δ*^15^N_col_ directly comparable to the plant isotopic data incorporated into the SIAR mixing model (Fig. 2).

The plant functional groupings (terrestrial trees and shrubs, emergent, floating, and submerged macrophytes) were statistically defined using scripts in SIBER in R Studio 3.1.2 (Figure B and C in Supplementary Information File SI1). The Total Area Convex Hull and Standard Ellipse plots aid in identifying the niche size and relative position of each plant functional group. The relative contribution of each plant functional group to the diet of each rodent species was assessed using scripts from SIAR V4 in R Studio 3.1.2 (Stable Isotope Analysis in R: An Ecologist’s Guide)^40^.

**Figure 3:**
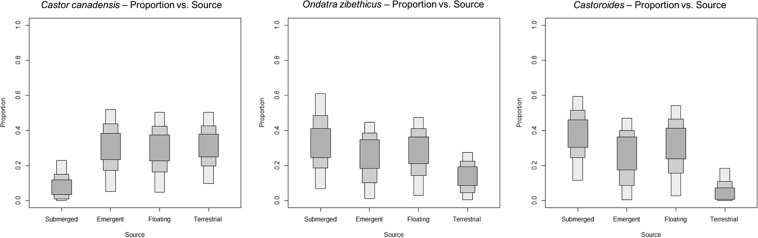
Proportion versus Source boxplots generated in SIAR for *C. canadensis*, *O. zibethicus*, and *Castoroides*. Each boxplot represents the relative proportion that each plant functional group (categorized here as a “Source”) contributed to the diet of each rodent species. The carbon isotope data for modern plants and rodents are corrected for the Suess effect.

The Proportion versus Source boxplot for *Castoroides* indicates that submerged and floating macrophytes were central to giant beaver diet, while terrestrial forage sources were utilized sparingly (Fig. 3; see Table C in Supplementary Information File SI1 for plant functional group mean and range). This pattern is a striking contrast to the Proportion versus Source boxplot for *C*. *canadensis* (Fig. 3). This suggests that the giant beaver and its smaller cousin had complementary forage preferences and were not in direct competition for the same food resources. Overall, submerged macrophytes appear to contribute a greater proportion to the diet of the giant beaver than they do to the diet of the extant muskrat or beaver.

The Proportion versus Source boxplot for *C*. *canadensis* (Fig. 3) indicates a diet composed primarily of terrestrial browse, and emergent and floating macrophytes. This correlates well with what is known about modern beaver diet (extensive consumption of tree foliage and floating macrophyte rhizomes)^62–64^.

The Proportion versus Source boxplot for *O*. *zibethicus* (Fig. 3) indicates a diet composed of approximately equal proportions of each macrophyte type, with a small proportion of terrestrial material. It appears that floating and submerged macrophytes are more integral to muskrat diet that previously reported^73,74^.

The *Castoroides δ*^13^C_col_ and *δ*^15^N_col_ support the hypothesis that the giant beaver was a semi-aquatic rodent that grazed predominantly on aquatic macrophytes. Our *δ*^13^C_col_ results are consistent with Boulanger *et al*.’s report of *Castoroides* (tooth dentin, n = 1) *δ*^13^C_col_ = −21.3‰^65^. Additional comparisons between modern and Pleistocene *Castor* and *Ondatr*a *δ*^13^C_col_ and *δ*^15^N_col_ would strengthen these conclusions. Unfortunately, there is very little published stable isotope data available from Pleistocene *Castor* and *Ondatra* specimens. There are isotopic data for a handful of samples (*Castor* n = 2; *Ondatra* n = 4) from the Aucilla River, Florida^75^; however, the bone collagen carbon and nitrogen contents indicates poor preservation, and hence the isotopic results may not record original compositions. Further analyses are required to make an effective comparison.

A strong preference for submerged macrophytes rendered *Castoroides* highly dependent on wetland habitat for sustenance. The Proportion versus Source boxplots generated in SIAR strongly suggest that *C*. *canadensis* and *Castoroides* occupied complementary dietary niches. While they likely competed for habitat space, their forage preferences were sufficiently different to allow the two genera to co-exist across North America during the mid- to late Pleistocene. When assessing C*astoroides*’ impact on the Pleistocene landscape, neither *C*. *canadensis* nor *O*. *zibethicus* constitute a perfect analogue species; however, based on *δ*^13^C_col_ and *δ*^15^N_col_ results, the muskrat is the closest extant semi-aquatic rodent that could be used to describe the diet and ecological impact of the giant beaver. These interpretations remain supported even if only *δ*^13^C_col_ are compared among the three rodent species (refer to Figure D in Supplementary Information File SI1).

**Figure 4:**
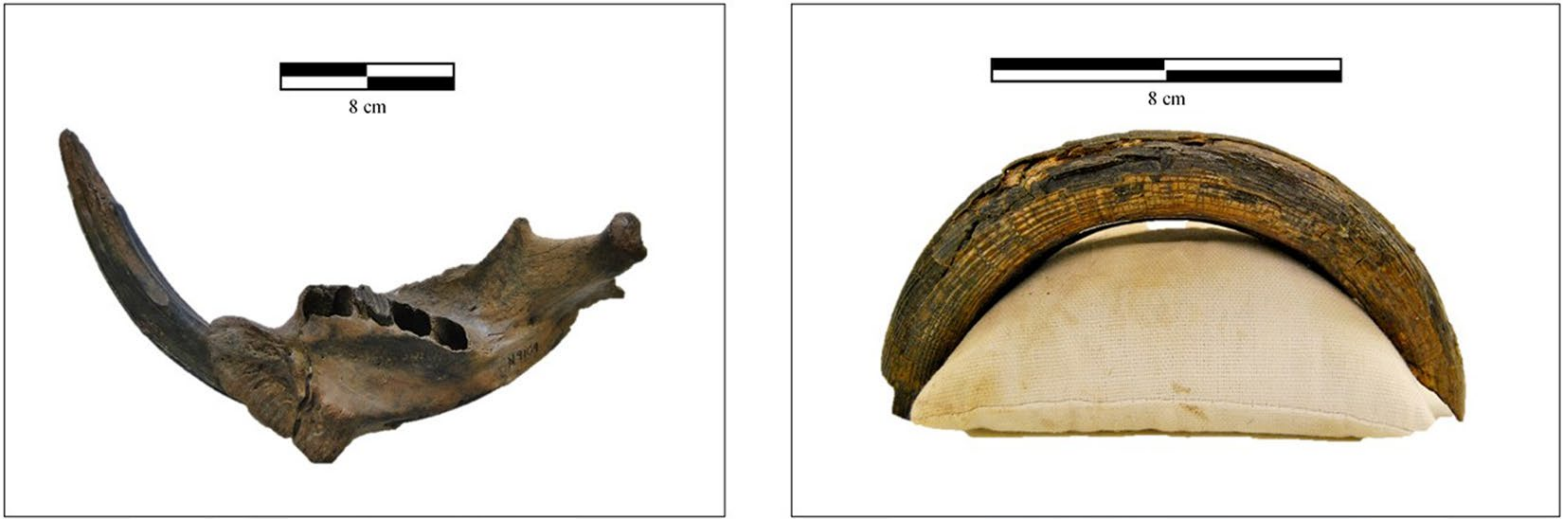
Left: A near-complete *Castoroides ohioensis* mandible and lower incisor from Clear Creek Township, Ohio, USA. Specimen OHS N9109. Right: A complete *Castoroides ohioensis* upper incisor from Old Crow, Yukon Territory, Canada. Specimen CMN 51270. Photographs by T. Plint.

The Proportion versus Source boxplots generated in SIAR do not lend support to the notion that *Castoroides* cut down trees or consumed foods such as tree leaves, bark or twigs. There is currently no convincing evidence of dams, lodges, or underwater food caches constructed by giant beavers from the Pleistocene sedimentological record. If the giant beaver was felling trees for food, the stable isotopic composition of its bone collagen should reflect a greater proportion of terrestrial browse (see Supplementary Information File SI1 Table C for plant functional group *δ*^13^C and *δ*^15^N means and ranges). This conclusion is further supported by skeletal morphology, in that giant beaver incisors lack the chisel-like edge that is characteristic of *Castor* incisors^76^ (Fig. 4). *C*. *canadensis*’ ability to engineer the landscape and to create new habitat space, combined with the rise in abundance and diversity of deciduous tree species in north-eastern North America, likely gave *Castor* a distinct advantage over the giant beaver during the late Pleistocene. Hence competition for habitat space may have been a contributing factor in the extinction of the giant beaver. Testing this hypothesis, however, awaits a suitable suite of fossil remains of coeval *Castor* and *Castoroides*.

Confirmation that *Castoroides* was highly dependent on wetland habitat not only for shelter from predators, but also for food allows us to assess its extinction within the context of changing environmental conditions during the late Pleistocene. The loss of both wetland habitat in lowland regions and associated open mixed-conifer forests coincide with the regional disappearance of *Castoroides* populations in many areas across North America, as is discussed next.

The first appearance of *Castoroides* in the fossil record is in Florida and dates to the Irvingtonian I NALMA (1.9 mya to 900,000 BP)^2,77,78^. There is a more or less continuous record of *Castoroides* in the southeast, where the oldest known species of giant beaver, *Castoroides leiseyorum*, is assumed to have given rise to both *Castoroides ohioensis* and *Castoroides dilophidus*^79^. Unlike *C*. *ohioensis*, it appears that *C*. *dilophidus* remained confined to the southeast (Florida, Georgia, South Carolina, and Virginia) during the Rancholabrean NALMA (240,000 to 11,000 year BP)^79,80^. Virtually all specimens from Florida have been collected from river systems and many lack precise contextual information^80^. An insecure faunal chronology, combined with scarce pollen and plant macrofossil records for the region prior to the Wisconsin Glaciation, limit our ability to discern ecological patterns over time. However, the region supported forested swamps and warm-adapted mixed forests during much of the mid-Wisconsin. Florida underwent multiple periods of increasing aridity and more seasonal precipitation patterns throughout the Last Glacial Maximum (approximately 24,000 years BP)^81^. Scrub and prairie conditions arose in parts of Georgia, the Coastal Plain, and in southern Florida during both Wisconsin glacial and post-glacial times^82^. Moister conditions that favoured pine forests and swampland did not return until mid- to late Holocene, long after the extinction of the giant beaver^81–83^.

The first records of *Castoroides* in the Great Plains region are from Kansas and date to the Irvingtonian NALMA. However, the transition from an ancestral taxon *Procastoroides* (uncrenulated incisors) to *Castoroides* (crenulated incisors) probably occurred on the Great Plains during either the late Pliocene or the early Pleistocene during the Blancan NALMA^2,84^. Giant beaver populations became well-established across Kansas, Nebraska, and Oklahoma during the late Irvingtonian and early Rancholabrean NALMA^1,80^. After onset of the Illinoian glaciation, giant beaver fossils disappeared from the Great Plains record and became concentrated east of the Mississippi River. The disappearance of no-analog forest communities, the regional extinction of certain conifer tree species, and the appearance of grass or herb plant communities all correlate to increasing aridity and coincide with the disappearance of the giant beaver in the region^85–87^.

In the north, *Castoroides* and *Castor* fossils are both found in lake bed and river deposits from the unglaciated region of Old Crow, Yukon Territory^88^. Giant beavers most likely inhabited Yukon Territory and Alaska during global warm periods between 200,000 to 75,000 years BP^89,90^. This chronology is similar to that of the American mastodon (*Mammut americanum*) and western camel (*Camelops hesternus*) and are interpreted to represent northward dispersals during the relatively warm Sangamonian interglaciation^49,91^. The presence of *Castoroides* fossils from *in situ* deposits correlative to Marine Isotope Stage 7 (~200,000 years BP) suggests there were repeated northward dispersals into the Arctic during at least two separate interglaciations^89^. Giant beaver populations could only have spread northward during these periods of warm climate, where the retreat of the ice sheets left a string of meltwater wetlands, and boreal forests and wet-tundra were established regionally. The presence of *Castoroides* in these arctic latitudes suggests they were capable of surviving the cold dark winters of interglacial periods, though regional aridification and establishment of cryoxerophilous steppe tundra vegetation probably created environments that were not habitable for them during glacial periods. It is highly likely that the giant beaver was regionally extirpated with the establishment of Wisconsinan glacial conditions around 70,000 years BP and subsequently all populations were restricted to areas south of the continental ice sheets until their final demise at the end of the Pleistocene^90^.

Radiocarbon dates from Ohio and New York indicate that the Great Lakes Basin was home to the last known population of giant beavers^65^. Fossils from the Sangamonian are also occasionally found. The population was concentrated south of the fluctuating continental ice sheet margin during the Wisconsinan glacial period prior to the extinction of the genus^1,17–19,65,66^. The giant beaver disappeared from Eastern North America shortly before the Pleistocene-Holocene transition^6^. With the disappearance of this population, the genus became globally extinct.

Wisconsinan age giant beaver fossils are more commonly associated with sediments from warmer interstadial times, when the retreat of continental ice sheets left the Great Lakes region flooded with meltwater. The cool, moist climate during the mid-Wisconsinan provided ideal habitat space in the form of extensive lacustrine environments surrounded by forest communities dominated by spruce and larch^19,58^. During the onset of the Holocene warm period, much of the shallow wetland habitat in Eastern North America became in-filled with sediment and deciduous trees replaced much of the open mixed-conifer forest communities^92^.

The only known instance of direct temporal and spatial overlap between human artifacts and *Castoroides* fossils occurs in New York State. The current youngest known *Castoroides* specimen (dating to 10,150 ± 50 years BP) indicates that megafauna populations overlapped with Palaeoindian culture for up to a thousand years^6^. However, there is no current zooarchaeological evidence that humans butchered, hunted, or otherwise utilized the giant beaver as a resource.

## Conclusions

*Castoroides δ*^13^C_col_ and *δ*^15^N_col_ indicate that giant beaver diet was composed predominantly of macrophytes (particularly submerged macrophytes), rendering the genus highly dependent on wetland habitat for survival. The presence of giant beaver fossils coincides with palaeoenvironmental conditions that supported ample swamps and shallow lakes, and such remains disappear in settings associated with the arrival of warmer, more arid conditions. Sediment infilling, loss of glacial meltwater, the onset of more seasonal precipitation patterns, and increased temperature all contributed to wetland habitat loss across North America during this time. Without apparent refugia, *Castoroides* dwindled to an isolated population located in the lowlands south of the Great Lakes, where potential competition for habitat space and ongoing profound climate change contributed to their extinction.

## Methodology

### Rodent sample material

*Castoroides* specimens originating from Yukon Territory, Canada, and Ohio, USA, were included to determine regional differences in diet (Fig. 1). Modern muskrat and beaver skeletal material were included to explore similarities in econiche and the accuracy of the SIAR mixing model. To facilitate comparison, the modern *O*. *zibethicus* and *C*. *canadensis* samples were sought from similar locations as the *Castoroides* fossils (Fig. 1, Tables 3 to 5). Modern skeletal materials were donated by conservation authorities, trappers, and ecological research groups.

### Radiocarbon dating

Radiocarbon (^14^C) dating of *Castoroides* bone samples collected in Yukon Territory was performed at the Keck Carbon Cycle AMS Facility, University of California Irvine. The Keck laboratory employed ultrafiltration of the gelatinized collagen at 30 kDa, and a second time at 3 kDa to remove exogenous organic material^93^. Radiocarbon dating of *Castoroides* bone samples collected in Ohio was performed by the Accelerator Mass Spectrometry Laboratory, University of Arizona using a modified Longin bulk collagen extraction method without ultrafiltration^94^.

### Collagen stable isotope analysis

Detailed information regarding collagen sample preparation and stable isotope analysis is provided in Supplementary Information File SI1. In brief, collagen was extracted using a modified Longin method^94^. Crushed sample material underwent lipid extraction, followed by slow demineralization in a weak acid, and a final soak in a weak base to remove humic acids. Gelatinized collagen was analyzed using a Costech Elemental Analyzer coupled with a Thermo Scientific Delta Plus XL isotope ratio mass spectrometer operated in continuous-flow mode with helium as the carrier gas. Stable carbon and nitrogen isotope results are reported relative to VPDB and AIR, respectively.

### Stable isotope analysis of plants

An assessment of bulk plant carbon and nitrogen isotope signatures was undertaken to provide the necessary context to interpret *Castoroides*, *C*. *canadensis* and *O*. *zibethicus δ*^13^C_col_ and *δ*^15^N_col_. Supplementary Information File SI1 provides detailed information regarding plant sample taxonomic identification, preparation, and stable isotope analysis performed for this purpose. We analyzed four plant functional groups: terrestrial trees and shrubs, emergent macrophytes, floating macrophytes, and submerged macrophytes. These groupings were chosen to (1) identify plants with access to similar pools of bioavailable carbon and nitrogen, and (2) to gauge *Castoroides*’ level of dependence on freshwater versus terrestrial food resources. Similar plant functional groupings are utilized in studies of modern *C*. *canadensis*^54,95^.

Modern plant samples were collected from shallow lakes, marshes, and creeks located in southwestern Ontario (Pinery Provincial Park and the London area) and Yukon Territory (Whitehorse and Old Crow Basin), Canada (Fig. 1).

The Ontario sites experience a temperate climate, and with annual average precipitation of approximately 1000 mm. Pinery Provincial Park is located on the south-east shore of Lake Huron. The park consists of the northern-most extension of the Oak Savannah ecosystem and overlays a succession of ancient dune ridges. London is located approximately 70 km east of Pinery Provincial Park, and is located in an area of Quaternary sediments (glacial till interbedded with interglacial lake sediments) overlaying Paleozoic limestone bedrock. The sites in Yukon Territory experience a subarctic climate, where annual average precipitation is between 200 and 300 mm. Whitehorse is located within the Whitehorse trough (an intermontane basin) in south-central Yukon Territory. The Old Crow community is located in a region of continuous permafrost within the Arctic Circle and is situated along the southern fringe of the Old Crow Flats (a plain filled with thermokarst lakes). The region is surrounded by adjacent mountain ranges and remained unglaciated during the Quaternary.

## Supplementary information

Supplementary Information File SI1

## Data Availability

The authors declare no limitations on data or standard operating protocol availability.
