# Author Correction: Giant beaver palaeoecology inferred from stable isotopes

**DOI:** 10.1038/s41598-020-61338-y

**Published:** 2020-03-06

**Authors:** Tessa Plint, Fred J. Longstaffe, Grant Zazula

**Affiliations:** 1 Department of Earth Sciences, The University of Western Ontario, London, Ontario Canada; 2 The Lyell Centre, School of Energy, Geoscience, Infrastructure and Society, Heriot-Watt University, Edinburgh, United Kingdom; 3 Yukon Palaeontology Program, Government of Yukon, Whitehorse, Yukon Territory Canada; 4 Research and Collections, Canadian Museum of Nature, PO Box 3443, Station D, Ottawa, ON K1P 6P4 Canada

Correction to: *Scientific Reports* 10.1038/s41598-019-43710-9, published online 09 May 2019

The Acknowledgements section in this Article is incomplete.

“The authors thank the following people for their assistance: Jane Bowles, Michael Burzynski, Mike Dorland, Li Huang, Kim Law, Natalia Rybczynski, Rachel Schwartz-Narbonne, Racel Sopoco, Farnoush Tahmasebi, Michelle Viglianti, and Grace Yau. We also thank the following institutions and individuals who provided sample material: Canadian Museum of Nature, Ohio Historical Society, Vuntut Gwitchin First Nation Government, Yukon Trappers Association, Long Point Conservation Authority, Wildlife Energetics and Ecology Lab (McGill University), Natasha Bumstead, Bill Fitzgerald, and Tom Porawski. This research was supported by funding from the following organizations: Natural Sciences and Engineering Research Council of Canada Discovery Grant (F.J.L.), The Faculty of Science (The University of Western Ontario) (T.P.), Alexander Graham Bell Canada Graduate Scholarship-Master’s (T.P.), Northern Training Grant from the Northern Scientific Training Program (T.P.), and Arcangelo Rea Family Foundation (T.P.). The L.S.I.S. infrastructure used in this research was funded in part by The Canada Foundation for Innovation (F.J.L.), Ontario Research Fund (F.J.L.), and Natural Sciences and Engineering Research Tools and Infrastructure grants (F.J.L.). Additional time for research (F.J.L.) was supported by the Canada Research Chairs Program. This is Laboratory for Stable Isotope Science (LSIS) Contribution #363.”

should read:

“The authors thank the following people for their assistance: Jane Bowles, Michael Burzynski, Mike Dorland, Ed Eastaugh, Lisa Hodgetts, Li Huang, Kim Law, Natalia Rybczynski, Rachel Schwartz-Narbonne, Racel Sopoco, Farnoush Tahmasebi, Michelle Viglianti, and Grace Yau. We also thank the following institutions and individuals who provided sample material: Canadian Museum of Nature, Ohio Historical Society, Vuntut Gwitchin First Nation Government, Yukon Trappers Association, Long Point Conservation Authority, Wildlife Energetics and Ecology Lab (McGill University), Natasha Bumstead, Bill Fitzgerald, and Tom Porawski. This research was supported by funding from the following organizations: Natural Sciences and Engineering Research Council of Canada Discovery Grant (F.J.L.), The Faculty of Science (The University of Western Ontario) (T.P.), Alexander Graham Bell Canada Graduate Scholarship-Master’s (T.P.), Northern Training Grant from the Northern Scientific Training Program (T.P.), and Arcangelo Rea Family Foundation (T.P.). The L.S.I.S. infrastructure used in this research was funded in part by The Canada Foundation for Innovation (F.J.L.), Ontario Research Fund (F.J.L.), and Natural Sciences and Engineering Research Tools and Infrastructure grants (F.J.L.). Additional time for research (F.J.L.) was supported by the Canada Research Chairs Program. This is Laboratory for Stable Isotope Science (LSIS) Contribution #363.”

