# Supplementary material for: Giant beaver palaeoecology inferred from stable isotopes
Source: Sci Rep. 2019 May 9;9:7179. doi: 10.1038/s41598-019-43710-9 (PMC6509321; doi:10.1038/s41598-019-43710-9)
Supplement: Supplementary file 1 — Supplementary Information File SI1 [file 41598_2019_43710_MOESM1_ESM.pdf]

Giant beaver palaeoecology inferred from stable isotopes

Tessa Plint, Fred J. Longstaffe, Grant Zazula

Collagen sample preparation and stable isotope analysis

Collagen extraction was performed using a modified Longin (1971) method. The extraction procedure was virtually identical for ancient and modern rodent skeletal samples. All *Castoroides* specimens were documented as adult individuals (based on size). Crushed bone and dentin samples (500 mg) underwent lipid extraction using a 2:1 solution of chloroform and methanol. Samples were then demineralized in 0.5 M hydrochloric acid (HCl) at room temperature. Demineralized samples were soaked in a 0.1 M sodium hydroxide (NaOH) solution to remove humic acids. Collagen was gelatinized in pH 3 0.25 M HCl at 90°C for 16 hours, and then desiccated at 90°C for 24 hours. Collagen samples were analyzed using a Costech Elemental Analyzer coupled with a Thermo Scientific Delta Plus XL isotope ratio mass spectrometer operated in continuous-flow mode with helium as the carrier gas. Stable carbon and nitrogen isotope results were calibrated to VPDB and AIR respectively, using international standards USGS-40 (δ^{13}C: ± 0.2 ‰ 1SD, n = 8, accepted –26.4 ‰; δ^{15}N: ± 0.1 ‰ 1SD, n = 8, accepted –4.5 ‰) and USGS-41 (δ^{13}C: ± 0.4 ‰ 1SD, n = 9, accepted +37.6 ‰; δ^{15}N: ± 0.1 ‰ 1SD, n = 9 accepted +47.6 ‰). Keratin powder (in-house standard, MP Biomedicals Inc., Cat No. 90211, Lot No. 9966H) and IAEA-CH-6 were analyzed as unknowns to test for accuracy and precision. Keratin measured δ^{13}C = –24.1 ± 0.1 ‰ (mean ± 1SD, n = 18; internally calibrated –24.1 ‰), and keratin measured δ^{15}N = +6.36 ± 0.1 ‰ (mean ± 1SD, n = 18; internally calibrated +6.4 ‰). IAEA-CH-6 was used in a similar fashion, where measured δ^{13}C = –10.5 ± 0.1 ‰ (mean ± 1SD, n = 7; accepted –10.45 ‰). A minimum of one in-house and one international standard was analyzed for every five samples. One methodological (*i.e.*, separate collagen isolation) duplicate and one sample (*i.e.*, re-analysis of same collagen) duplicate were included for every ten samples analyzed. Reproducibility between duplicates was ± 0.3 ‰ for δ^{13}C and ± 0.1 ‰ for δ^{15}N.

Plant sample preparation and stable isotope analysis

Terrestrial and macrophyte plant samples were collected from four different Canadian localities: Old Crow Basin, Yukon Territory; Whitehorse, Yukon Territory; Pinery Provincial Park, Ontario; and the London area, Ontario (Figure 1). Plant samples from Yukon Territory and London, Ontario were collected in August 2014. Aquatic macrophytes from Pinery Provincial Park were collected in August 2014, whereas terrestrial trees and shrubs were collected in August 2000. All plant sample collection sites were chosen from areas with minimal documented or perceived human impact.

After collection, plant samples were air-dried for a minimum of five days at room temperature. Subsamples were removed from the original specimen and washed in distilled water using a soft brush and fine mesh sieve. Subsamples were sonicated for ~60 seconds to remove adhering algae or debris. Washed subsamples were dried overnight at 90°C before being crushed into a fine powder using a ball mill. Then, sample powder was weighed into tin capsules for concurrent carbon isotope analysis, and carbon and nitrogen contents. These concurrent analyses allowed optimum sample weights to be recalculated based on individual plant nitrogen contents. New sample aliquots of appropriate weight were then analyzed for nitrogen isotope composition.

Analyses were performed using a Costech Elemental Analyzer coupled to a Thermo Scientific Delta Plus XL isotope ratio mass spectrometer operated in continuous-flow mode, using helium as the carrier gas. Values of δ^{13}C were calibrated to VPDB using USGS-40 (± 0.1 ‰ 1SD, n = 19, accepted –26.4‰) and USGS-41 (± 0.1 ‰ 1SD, n = 20, accepted +37.6 ‰,). Keratin powder (in-house standard, MP Biomedicals Inc., Cat No. 90211, Lot No. 9966H) and IAEA-CH-6 were used to track analytical precision and accuracy. Keratin measured δ^{13}C = –24.0 ± 0.1 ‰ (mean ± 1SD, n = 38; internally calibrated –24.0 ‰). IAEA-CH-6 measured δ^{13}C = –10.5 ± 0.1 ‰ (mean ± 1SD, n = 14, accepted –10.45 ‰).

Values of δ^{15}N for the nitrogen-only analytical sessions were calibrated to AIR using USGS-40 (± 0.1 SD, n = 11; accepted –4.5 ‰) and USGS-41 (± 0.2 ‰ SD, n = 15; accepted +47.6 ‰). NIST-1547 (Peach leaves) and keratin powder were used as in-house standards to track analytical precision and accuracy. Keratin measured δ^{15}N = +6.5 ± 0.1 ‰ (mean ± 1SD, n = 31, internally calibrated +6.4 ‰). NIST-1547 measured δ^{15}N = +2.0 ± 0.1 ‰ (mean ± 1SD, n = 11, internally

calibrated +1.98 ‰). Analytical error for concurrent carbon and nitrogen, and N-only isotope measurements was ± 0.2 ‰. A minimum of one in-house and one international standard was analyzed for every five samples. One sample duplicate was included for every ten samples analyzed. Reproducibility between duplicates was ± 0.3 ‰ for δ^{13}C and ± 0.1 ‰ for δ^{15}N.

Table A. Taxonomic identification and stable isotope results for plant samples collected in Yukon Territory, Canada (August, 2014). Plant taxonomic identification was completed using photographs from the field. The Yukon Territory lichen and terrestrial moss carbon and nitrogen isotope data were not used in the SIAR mixing model. The carbon isotope data listed below are not corrected for the Suess effect.

Project sample ID	Taxon	Common name	Sample origin	Functional group	Sample type	δ^{13}C (‰, VPDB)	δ^{15}N (‰, AIR)
TP005	*Utricularia sp.*	Bladderwort	Old Crow	submerged	bulk foliage	–29.7	+4.1
TP007	*Potamogeton*	Pond weed	Old Crow	submerged	bulk foliage	–15.2	–2.7
TP008	?	Aquatic moss	Old Crow	submerged	bulk foliage	–31.4	+0.5
TP010	*Utricularia sp.*	Bladderwort	Old Crow	submerged	bulk foliage	–29.6	+5.1
TP011	*Potamogeton*	Pond weed	Old Crow	submerged	entire plant	–20.9	+0.1
TP012A	*Myriophyllum*	Water milfoil	Old Crow	submerged	bulk foliage	–14.2	–0.9
TP016	*Hippurus*	Mare's tail	Old Crow	submerged	bulk foliage	–30.6	+3.0
TP017A	*Hippurus*	Mare's tail	Old Crow	submerged	bulk foliage	–32.4	+0.1
TP026A	?	Pond weed	Old Crow	submerged	bulk foliage	–30.6	+1.2
TP030	?	Pond weed	Old Crow	submerged	entire plant	–26.9	+0.6
TP031	*Hippurus*	Mare's tail	Old Crow	submerged	bulk foliage	–32.3	+0.1
TP032	*Hippurus*	Mare's tail	Old Crow	submerged	bulk foliage	–30.4	+2.3
TP034	?	Aquatic moss	Old Crow	submerged	bulk foliage	–36.1	+0.8
TP035	*Potamogeton*	Pond weed	Old Crow	submerged	bulk foliage	–23.2	–2.0
TP051A	*Myriophyllum sp.*	Water milfoil	Whitehorse	submerged	bulk foliage	–13.1	+4.9
TP052A	*Potamogeton sp.*	Pond weed	Whitehorse	submerged	bulk foliage	–14.8	+3.8
TP052B	*Potamogeton sp.*	Pond weed	Whitehorse	submerged	bulk foliage	–14.8	+3.1
TP062	?	Pond weed	Whitehorse	submerged	bulk foliage	–28.1	+5.5
TP089	*Myriophyllum sp.*	Water milfoil	Whitehorse	submerged	bulk foliage	–41.2	+2.2

TP002	*Menyanthes trifoliata*	Buckbean	Old Crow	emergent	bulk foliage	–30.4	+1.3
TP013A	*Carex*	Sedge	Old Crow	emergent	bulk foliage	–30.3	+0.9
TP015	*Equisetum fluviatile*	Swamp horsetail	Old Crow	emergent	bulk foliage	–27.1	+2.5
TP025A	*Menyanthes trifoliata*	Buckbean	Old Crow	emergent	bulk foliage	–26.8	+1.8
TP027	*Equisetum pratense*	Shade horsetail	Old Crow	emergent	bulk foliage	–29.6	+3.7
TP053A	*Carex sp.*	Sedge	Whitehorse	emergent	base of stem	–27.8	+5.9
TP053B	*Carex sp.*	Sedge	Whitehorse	emergent	bulk leaves & seeds	–27.9	+6.4
TP056	?	Grass	Whitehorse	emergent	bulk foliage	–30.7	+4.7
TP057	*Equisetum*	Horsetail	Whitehorse	emergent	bulk foliage	–27.5	+6.6
TP059A	*Sparganium sp.*	Bur–reed	Whitehorse	emergent	bulk foliage & seeds	–28.2	+4.8
TP060A	*Carex sp.*	Sedge	Whitehorse	emergent	bulk foliage & seeds	–30.1	+4.8
TP060B	*Carex sp.*	Sedge	Whitehorse	emergent	base of stem	–28.3	+5.0
TP061	*Sparganium sp.*	Bur–reed	Whitehorse	emergent	bulk foliage	–28.5	+4.6
TP074A	*Carex sp.*	Sedge	Whitehorse	emergent	bulk foliage	–26.3	+5.1
TP074B	*Carex sp.*	Sedge	Whitehorse	emergent	base of stem	–25.6	+4.5
TP088	*Hippurus*	Mare's tail	Whitehorse	emergent	bulk foliage	–35.3	+4.0
TP018	*Lemna*	Duckweed	Old Crow	floating	entire plant	–31.6	+2.7
TP050	*Lemna trisculca*	Duckweed	Whitehorse	floating	entire plant	–23.3	+5.4
TP028A	*Vaccinium vitis–idaea*	Lingonberry	Old Crow	shrub	bulk foliage	–29.5	–1.6
TP043A	*Picea glauca*	White spruce	Old Crow	tree	bulk foliage	–28.7	–8.5
TP043B	*Picea glauca*	White spruce	Old Crow	tree	wood	–24.3	–6.8
TP045A	*Alnus viridis subsp. crispa*	Mountain alder	Old Crow	tree	bulk foliage	–28.6	–1.4
TP046A	*Salix sp.*	Willow	Old Crow	tree	bulk foliage	–29.5	+2.6
TP046B	*Salix sp.*	Willow	Old Crow	tree	wood	–29.2	+1.1

TP047	*Salix sp.*	Willow	Old Crow	tree	bulk foliage	–28.9	0.0
TP048A	*Alnus viridis subsp. crispa*	Mountain alder	Old Crow	tree	bulk foliage	–28.3	–1.2
TP048B	*Alnus viridis subsp. crispa*	Mountain alder	Old Crow	tree	wood	–27.5	–1.5
TP049	*Betula neoalaskana*	Alaskan birch	Old Crow	tree	bulk foliage	–29.8	–5.0
TP063A	*Salix sp.*	Willow	Whitehorse	tree	wood	–30.5	–0.9
TP063B	*Salix sp.*	Willow	Whitehorse	tree	bark	–32.0	+0.3
TP064A	*Salix sp.*	Willow	Whitehorse	tree	bulk foliage	–29.5	+0.4
TP064B	*Salix sp.*	Willow	Whitehorse	tree	wood	–27.9	–0.5
TP065A	*Viburnum edule*	Highbush cranberry	Whitehorse	shrub	bulk foliage	–31.1	–3.7
TP066	*Shepherdia canadensis*	Buffalo berry	Whitehorse	shrub	bulk foliage	–30.8	+0.1
TP068	*Populus tremuloides*	Aspen	Whitehorse	tree	bulk foliage	–31.3	–1.3
TP071	*Picea glauca*	White spruce	Whitehorse	tree	bulk foliage	–27.5	–1.3
TP077	*Salix sp.*	Willow	Whitehorse	tree	bulk foliage	–28.5	+3.9
TP081	*Betula glandulosa*	Shrub birch	Whitehorse	tree	bulk foliage	–30.3	–1.5
TP082	*Rhododendron groenlandicum*	Labrador tea	Whitehorse	shrub	bulk foliage	–28.5	–2.5
TP087	*Pinus contorta*	Lodgepole pine	Whitehorse	tree	bulk foliage	–27.7	–2.1
TP029	*?*	Moss	Old Crow	terrestrial	bulk foliage	–33.2	–1.1
TP036A	*?*	Lichen	Old Crow	terrestrial	entire plant	–24.8	–4.0
TP039	*Peltigera aphthosa* or *leucophlebia*	Dog tooth lichen	Old Crow	terrestrial	entire plant	–33.9	–0.5
TP086	*Peltigera leucophlebia*	Dog tooth lichen	Whitehorse	lichen	bulk plant	–35.1	–0.2

Table B. Taxonomic identification and stable isotope results for plant samples collected in Ontario, Canada (macrophyte samples collected August, 2014; woody plant samples collected August, 2000). Macrophyte taxonomic identification was completed using photographs from the field. The carbon isotope data listed below are not corrected for the Suess effect.

Project sample ID	Taxon	Common name	Sample origin	Functional group	Sample type	δ^{13}C (‰, VPDB)	δ^{15}N (‰, AIR)
TP091A	*Myriophyllum*	Water milfoil	Pinery Provincial Park	submerged	bulk foliage	–18.6	–1.1
TP093	*Utricularia sp*	Bladderwort	Pinery Provincial Park	submerged	bulk foliage	–29.0	+2.4
TP095	*Nitella sp.*	Calcified algae	Pinery Provincial Park	submerged	bulk foliage	–19.2	–3.3
TP099	*Myriophyllum*	Water milfoil	Pinery Provincial Park	submerged	bulk foliage	–23.9	–3.1
TP101A	*Potamogeton sp.*	Pond weed	Pinery Provincial Park	submerged	bulk foliage	–13.1	+2.8
TP101B	*Potamogeton sp.*	Pond weed	Pinery Provincial Park	submerged	bulk foliage	–12.9	+3.1
TP104	*Utricularia macrorhiza*	Bladderwort	Pinery Provincial Park	submerged	bulk foliage	–29.9	+1.3
TP107	*Heteranthera dubia*	Water stargrass	Pinery Provincial Park	submerged	bulk foliage	–13.9	–3.5
TP108	*Myriophyllum*	Water milfoil	Pinery Provincial Park	submerged	bulk foliage	–19.3	–3.1
TP110	*Potamogeton sp.*	Pond weed	Pinery Provincial Park	submerged	bulk foliage	–16.4	–1.5
TP116	*?*	?	London Area	submerged	bulk foliage	–23.4	+1.4
TP096A	*Bidens (possibly cernua)*	Aster	Pinery Provincial Park	emergent	bulk leaves & flowers	–30.2	+1.3
TP097A	*?*	Grass	Pinery Provincial Park	emergent	bulk foliage	–28.9	+0.2
TP097B	*?*	Grass	Pinery Provincial Park	emergent	base of stem	–28.8	+0.1
TP098	*?*	Grass	Pinery Provincial Park	emergent	bulk foliage	–29.3	+4.2
TP112	*Typha*	Bulrush	London Area	emergent	base of stem	–31.2	+1.8
TP113	*Typha*	Bulrush	London Area	emergent	bulk cattail	–28.3	+4.3
TP114	*?*	Arrowhead	London Area	emergent	bulk foliage	–30.7	+1.6

TP117A	*Typha*	Bulrush	London Area	emergent	bulk foliage	–31.1	+1.8
TP094	*Lemna sp.*	Duckweed	Pinery Provincial Park	floating	entire plant	–28.4	+1.0
TP102	*Potamogeton sp.*	Pond weed	Pinery Provincial Park	floating	bulk foliage	–25.7	+0.1
TP103	*Nuphar variegata*	Pond lily	Pinery Provincial Park	floating	bulk flower	–24.7	+1.5
TP111A	*?*	Arrowhead	London Area	floating	bulk foliage	–28.3	+7.2
TP118	*Nuphar*	Pond lily	London Area	floating	bulk foliage	–26.2	+6.3
TP119A	*Populus*	Aspen	London Area	tree	bulk foliage	–27.6	–0.8
TP119B	*Populus*	Aspen	London Area	tree	wood	–25.7	–1.2
VEG T O–B AUG1/00#1A	*Juniperus virginiana*	Red cedar	Pinery Provincial Park	tree	bulk foliage	–27.1	–3.5
VEG T O–B AUG1/00#1B	*Juniperus virginiana*	Red cedar	Pinery Provincial Park	tree	bulk twig	–26.5	–3.1
VEG S O–B AUG1/00#1A	*Populus balsamifera*	Balsam popular	Pinery Provincial Park	shrub	bulk foliage	–28.3	–4.2
VEG S O–B AUG1/00#1B	*Populus balsamifera*	Balsam popular	Pinery Provincial Park	shrub	bulk twig	–26.6	–6.4
VEG S O–B AUG1/00#2A	*Juniperus communis*	Common juniper	Pinery Provincial Park	shrub	bulk foliage	–25.9	–5.3
VEG S O–B AUG1/00#2B	*Juniperus communis*	Common juniper	Pinery Provincial Park	shrub	bulk twig	–23.9	–5.8
VEG S O–B AUG1/00#3A	*Prunus serotinia*	Black cherry	Pinery Provincial Park	shrub	bulk foliage	–27.8	–2.0
VEG S O–B AUG1/00#3B	*Prunus serotinia*	Black cherry	Pinery Provincial Park	shrub	bulk twig	–26.7	–2.4
VEG S O–A AUG3/00#1A	*Juniperus communis*	Common juniper	Pinery Provincial Park	shrub	bulk foliage	–25.7	–6.6
VEG S O–A AUG3/00#1B	*Juniperus communis*	Common juniper	Pinery Provincial Park	shrub	bulk twig	–24.4	–6.0
VEG S O–A AUG3/00#2A	*Populus balsamifera*	Balsam popular	Pinery Provincial Park	shrub	bulk foliage	–26.9	–7.5
VEG S O–A AUG3/00#3A	*Arctostaphylos uva ursi*	Bear berry	Pinery Provincial Park	shrub	bulk foliage	–28.5	–9.5
VEG S O–A AUG3/00#3B	*Arctostaphylos uva ursi*	Bear berry	Pinery Provincial Park	shrub	bulk twig	–26.6	–10.6

VEG T1AUG11/00#1A	*Prunus serotina*	Black cherry	Pinery Provincial Park	tree	bulk foliage	–28.5	–4.7
VEG T1AUG11/00#1B	*Prunus serotina*	Black cherry	Pinery Provincial Park	tree	bulk twig	–28.4	–3.8
VEGT1AUG11/00#2A	*Juniperus virginiana*	Red cedar	Pinery Provincial Park	tree	bulk foliage	–28.5	–5.1
VEGT1AUG11/00#2B	*Juniperus virginiana*	Red cedar	Pinery Provincial Park	tree	bulk twig	–27.2	–3.8
VEGT1AUG11/00#3A	*Quercus velutina*	Black oak	Pinery Provincial Park	tree	bulk foliage	–28.5	–5.9
VEGT1AUG11/00#3B	*Quercus velutina*	Black oak	Pinery Provincial Park	tree	bulk twig	–26.9	–5.1
VEGT1AUG11/00#4A	*Pinus strobus*	White pine	Pinery Provincial Park	tree	bulk foliage	–30.7	–3.7
VEGT1AUG11/00#4B	*Pinus strobus*	White pine	Pinery Provincial Park	tree	bulk twig	–30.1	–5.2
VEGT1AUG11/00#5A	*Quercus prinoides*	Chinquapin oak	Pinery Provincial Park	tree	bulk foliage	–28.5	–4.0
VEGT1AUG11/00#5B	*Quercus prinoides*	Chinquapin oak	Pinery Provincial Park	tree	bulk twig	–28.1	–4.3
VEGS1AUG3/00#2A	*Symphoricarpos duham*	Snowberry	Pinery Provincial Park	shrub	bulk foliage	–30.3	–7.6
VEGS1AUG3/00#2B	*Symphoricarpos duham*	Snowberry	Pinery Provincial Park	shrub	bulk twig	–29.8	–8.5
VEGT2AUG16/00#1A	*Pinus strobus*	White pine	Pinery Provincial Park	tree	bulk foliage	–29.0	–3.3
VEGT2AUG16/00#1B	*Pinus strobus*	White pine	Pinery Provincial Park	tree	bulk twig	–27.7	–4.0
VEGT2AUG16/00#2A	*Quercus velutina*	Black oak	Pinery Provincial Park	tree	bulk foliage	–27.8	–4.6
VEGT2AUG16/00#2B	*Quercus velutina*	Black oak	Pinery Provincial Park	tree	bulk twig	–27.8	–4.9
VEGT2AUG16/00#4A	*Prunus serotina*	Black cherry	Pinery Provincial Park	tree	bulk foliage	–29.5	–5.7
VEGT2AUG16/00#4B	*Prunus serotina*	Black cherry	Pinery Provincial Park	tree	bulk twig	–28.7	–5.4
VEGT2AUG16/00#5A	*Prunus virginiana*	Choke cherry	Pinery Provincial Park	tree	bulk foliage	–29.9	–2.5
VEGT2AUG16/00#5B	*Prunus virginiana*	Choke cherry	Pinery Provincial Park	tree	bulk twig	–29.9	–4.1
VEGS2AUG16/00#1A	*Symphoricarpos duham*	Snowberry	Pinery Provincial Park	shrub	bulk foliage	–30.7	–5.2
VEGT5AUG21/00#1A	*Pinus strobus*	White pine	Pinery Provincial Park	tree	bulk foliage	–27.9	–4.7
VEGT5AUG21/00#1B	*Pinus strobus*	White pine	Pinery Provincial Park	tree	bulk twig	–27.2	–5.8
VEGT5AUG21/00#3A	*Quercus alba*	White oak	Pinery Provincial Park	tree	bulk foliage	–29.7	–5.0

VEGT5AUG21/00#3B	*Quercus alba*	White oak	Pinery Provincial Park	tree	bulk twig	–30.1	–5.9
VEGS5AUG21/00/#1A	*Rhus aromatica*	Fragrant sumac	Pinery Provincial Park	shrub	bulk foliage	–29.2	–7.8
VEGS5AUG21/00#2A	*Vaccinium*	Blueberry	Pinery Provincial Park	shrub	bulk foliage	–30.7	–4.7
VEGS5AUG21/00#2B	*Vaccinium*	Blueberry	Pinery Provincial Park	shrub	bulk twig	–30.1	–4.6
VEGS5AUG21/00#3A	*Rubus*	Raspberry	Pinery Provincial Park	shrub	bulk foliage	–30.6	–5.1
VEGS5AUG21/00#3B	*Rubus*	Raspberry	Pinery Provincial Park	shrub	bulk twig	–30.5	–5.4
VEGS5AUG21/00#6A	*Prunus virginiana*	Choke cherry	Pinery Provincial Park	shrub	bulk foliage	–29.1	–6.2
VEGS.5AUG03/00#2A	*Populus balsamifera*	Balsam popular	Pinery Provincial Park	shrub	bulk foliage	–29.8	–4.5
VEGS.5AUG03/00#2B	*Populus balsamifera*	Balsam popular	Pinery Provincial Park	shrub	bulk twig	–27.4	–5.4

Table C. Summary of plant functional group $\delta^{13}C$ and $\delta^{15}N$.

Plant functional group	(n)	$\delta^{13}C_{mean}$ (‰, VPDB)	$\delta^{13}C_{range}$ (‰, VPDB)	$\delta^{15}N_{mean}$ (‰, AIR)	$\delta^{15}N_{range}$ (‰, AIR)
Yukon Territory sites					
Submerged	(19)	–26.1	–41.2 to –13.1	+1.7	–2.7 to +5.5
Emergent	(16)	–28.8	–35.3 to –25.6	+4.2	+0.9 to +6.6
Floating	(2)	–27.5	–31.6 to –23.3	+4.1	+2.7 to +5.4
Terrestrial	(22)	–29.1	–32.0 to –24.3	–1.4	–8.5 to +3.9
Ontario sites					
Submerged	(11)	–20.0	–29.2 to –12.9	–0.4	–3.5 to +3.1
Emergent	(8)	–29.8	–31.2 to –28.3	+1.9	+0.1 to +4.3
Floating	(5)	–26.6	–28.4 to –24.7	+3.2	+0.1 to +7.2
Terrestrial	(48)	–28.3	–30.7 to –29.9	–5.0	–10.6 to –0.8

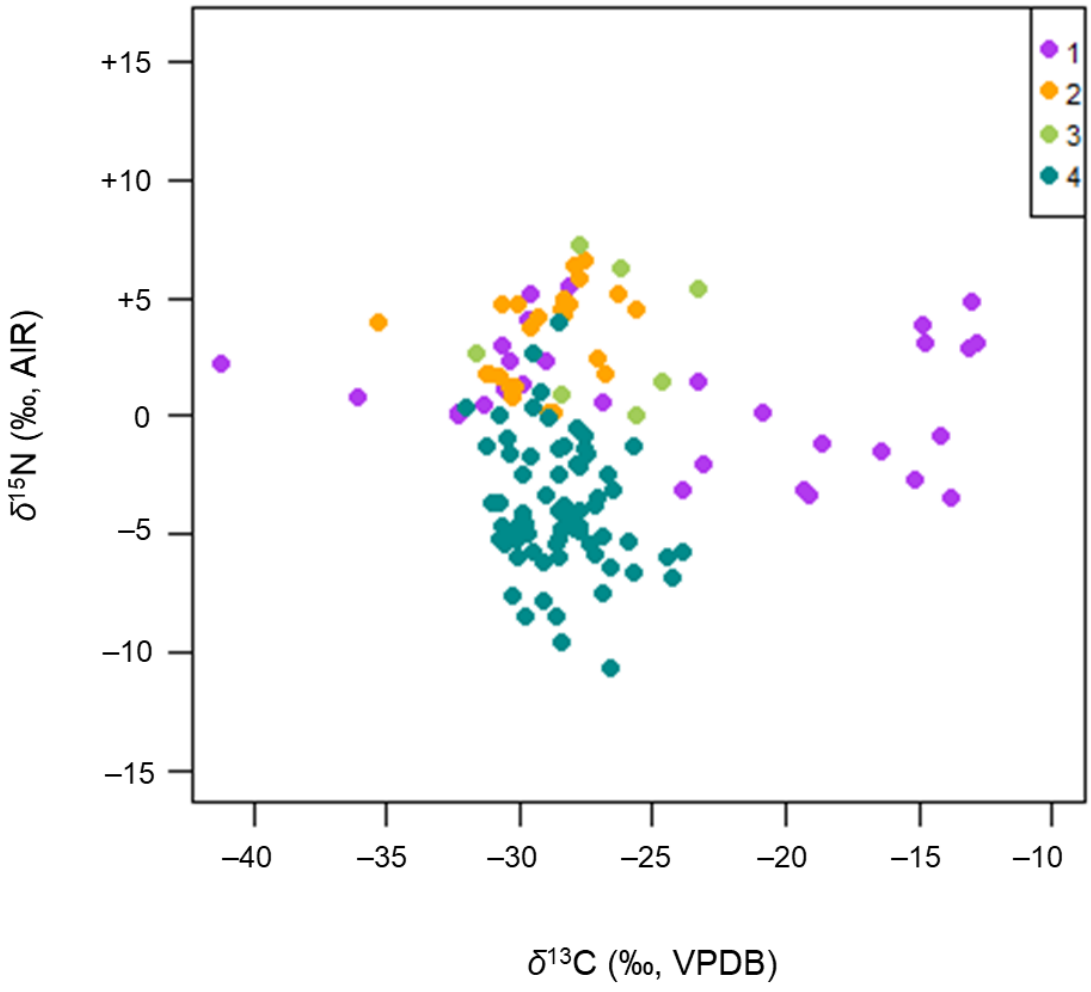

Figure A. Bivariate plot generated in SIBER for plant δ^{13}C and δ^{15}N. 1 – Submerged macrophytes. 2 – Emergent macrophytes. 3 – Floating macrophytes. 4 – Terrestrial trees and shrubs.

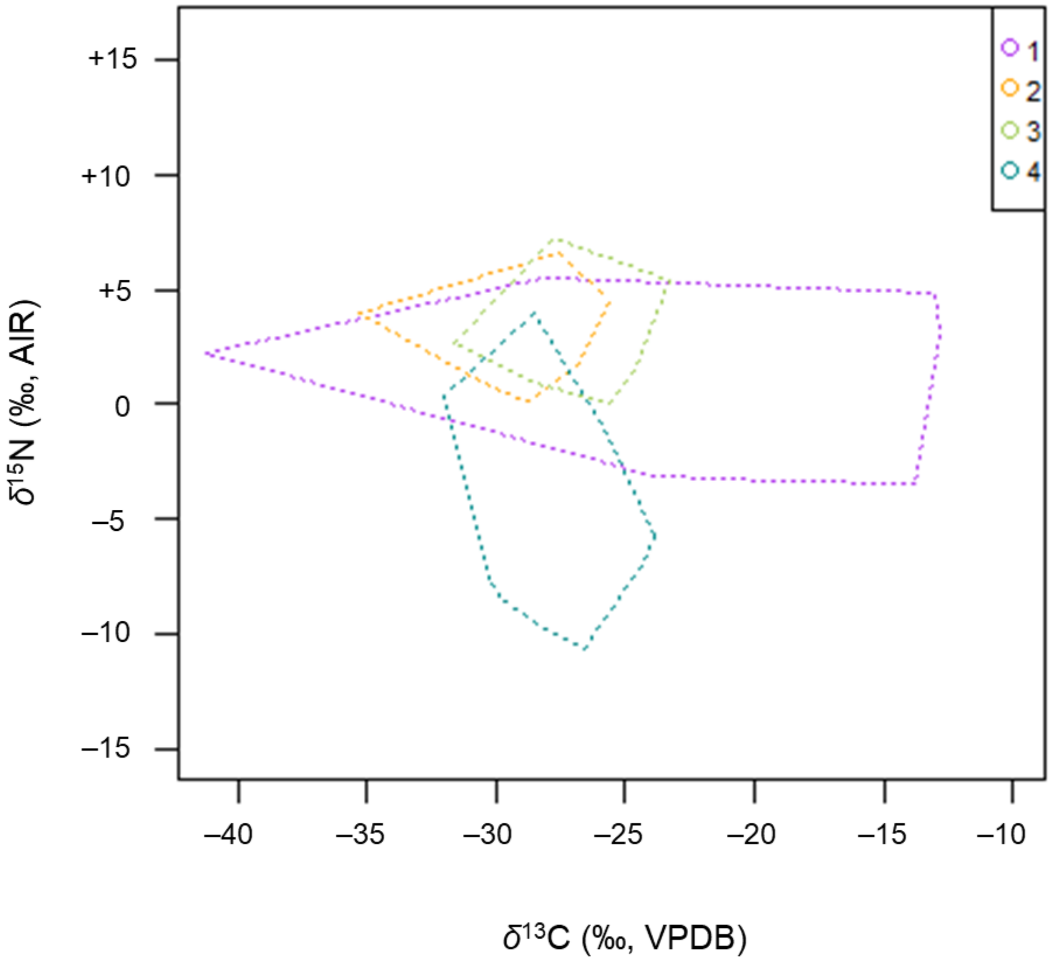

Figure B. Total Area Convex Hulls generated in SIBER for plant isotopic composition. The area of each hull denotes the size of the isotopic niche for each plant functional group. 1 – Submerged macrophytes. 2 – Emergent macrophytes. 3 – Floating macrophytes. 4 – Terrestrial trees and shrubs.

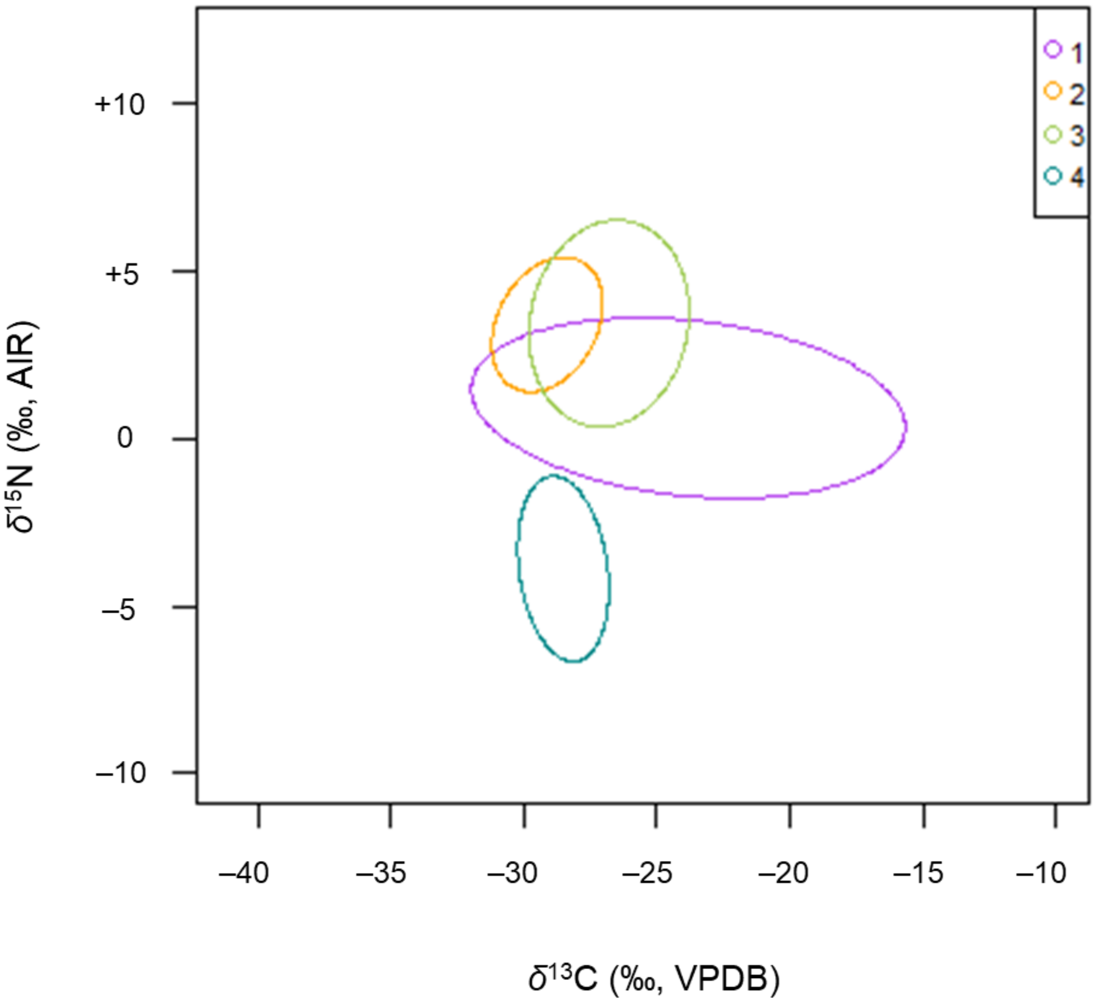

Figure C. Small Sample Size Corrected Standard Ellipses generated in SIBER for plant isotopic composition. Each ellipse displays 40% of the data per plant functional group. 1 – Submerged macrophytes. 2 – Emergent macrophytes. 3 – Floating macrophytes. 4 – Terrestrial trees and shrubs.

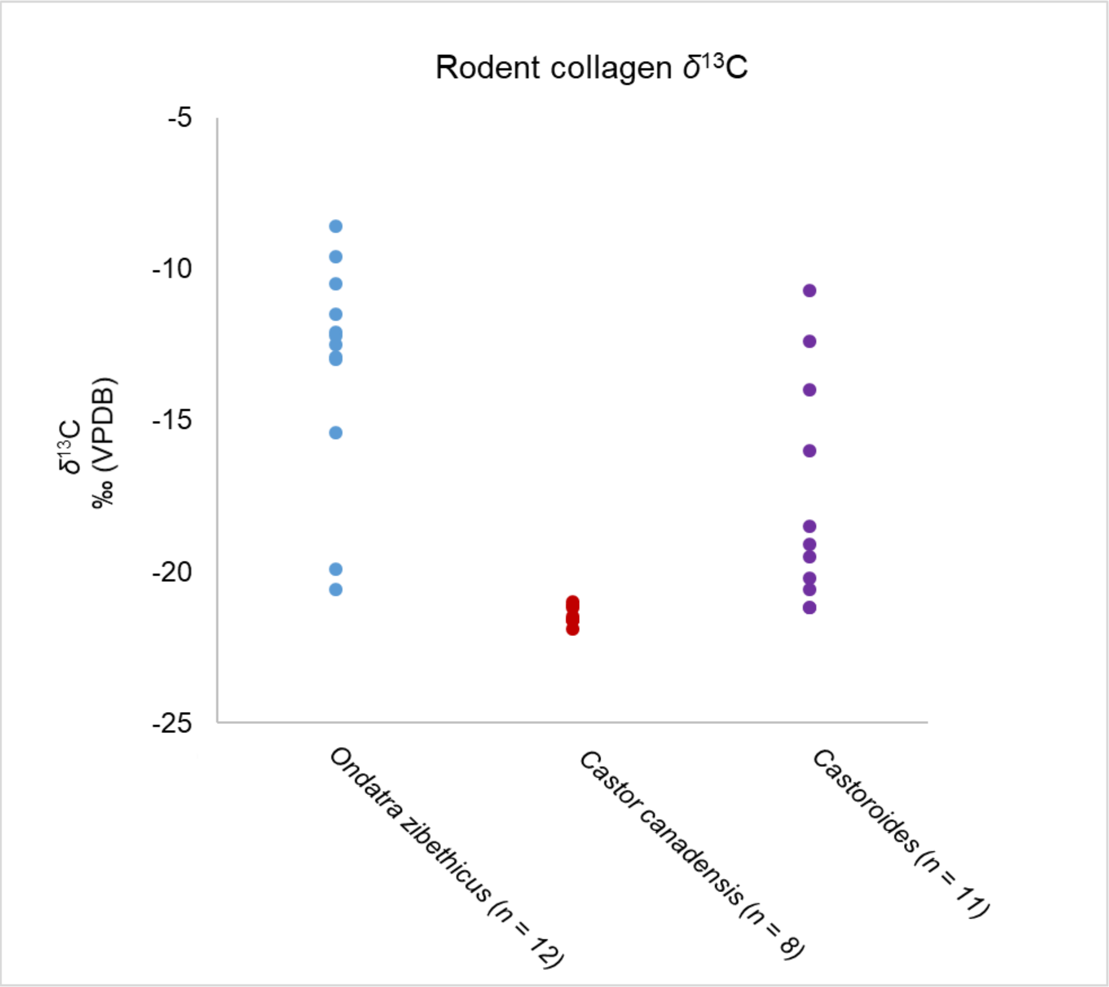

Figure D. Semi-aquatic rodent $\delta^{13}C_{col}$ (this study) supports the interpretation that *Castoroides* consumed ^{13}C-enriched macrophytes (noting that macrophytes have a very wide range of δ^{13}C) and that *Castor canadensis* consumed more ^{13}C-depleted terrestrial plant resources (noting that trees and shrubs have a narrow range of low δ^{13}C). Modern *Ondatra zibethicus* and *Castor canadensis* collagen $\delta^{13}C_{col}$ are corrected for the Suess effect.

References

Longin, R. New method of collagen extraction for radiocarbon dating. *Nature* **230**, 241 (1971).
